# Interim result of phase II, prospective, single-arm trial of long-course chemoradiotherapy combined with concurrent tislelizumab in locally advanced rectal cancer

**DOI:** 10.3389/fonc.2023.1057947

**Published:** 2023-02-02

**Authors:** Jiale Gao, Xiao Zhang, Zhengyang Yang, Jie Zhang, Zhigang Bai, Wei Deng, Guangyong Chen, Rui Xu, Qi Wei, Yishan Liu, Jiagang Han, Ang Li, Gang Liu, Yi Sun, Dalu Kong, Hongwei Yao, Zhongtao Zhang

**Affiliations:** ^1^ Department of General Surgery, Beijing Friendship Hospital, Capital Medical University & National Clinical Research Center for Digestive Diseases, Beijing, China; ^2^ Department of Radiology, Beijing Friendship Hospital, Capital Medical University, Beijing, China; ^3^ Department of Pathology, Beijing Friendship Hospital, Capital Medical University, Beijing, China; ^4^ Department of General Surgery, Beijing Chaoyang Hospital, Capital Medical University, Beijing, China; ^5^ Department of General Surgery, Xuanwu Hospital, Capital Medical University, Beijing, China; ^6^ Department of General Surgery, Tianjin Medical University General Hospital, Tianjin, China; ^7^ Department of Anorectal, Tianjin People’s Hospital, Tianjin, China; ^8^ Department of Colorectal Cancer, Key Laboratory of Cancer Prevention and Therapy of Tianjin, Tianjin’s Clinical Research Center for Cancer, National Clinical Research Center for Cancer, Tianjin Medical University Cancer Institute and Hospital, Tianjin, China

**Keywords:** rectal cancer, chemoradiotherapy, immune checkpoint inhibitors, neoadjuvant therapy, combination therapy

## Abstract

**Background:**

Neoadjuvant chemoradiotherapy is the standard treatment for locally advanced rectal cancer, with modest benefits on tumor regression and survival. Since chemoradiotherapy combined with immune checkpoint inhibitors has been reported to have synergic effects. This study aims to explore the safety and efficacy of long-course chemoradiotherapy combined with concurrent tislelizumab as a neoadjuvant treatment regimen for patients with locally advanced rectal cancer.

**Methods:**

This manuscript reported the interim result of a prospective, multicenter, single-arm, phase II trial. Patients with mid-to-low locally advanced rectal cancer with clinical stages of cT3-4a N0M0 or cT1-4a N1-2M0 were included. The patients received long-course radiotherapy (50 Gy/25 f, 2 Gy/f, 5 days/week) and three 21-day cycles of capecitabine (1000 mg/m2, bid, day1-14) plus concurrent three 21-day cycles of tislelizumab (200 mg, day8), followed by a radical surgery 6-8 weeks after radiotherapy. The primary endpoint was the pathological complete response rate. (Clinical trial number: NCT04911517)

**Results:**

A total of 26 patients completed the treatment protocol between April 2021 and June 2022. All patients completed chemoradiotherapy, 24 patients received three cycles of tislelizumab, and 2 patients received two cycles. The pathological complete remission (ypT0N0) was achieved in 50% (13/26) of the patients with all proficient mismatch repair tumors. The immune-related adverse event occurred in 19.2% (5/26) of patients. Patients with no CEA elevation or age less than 50 were more likely to benefit from this treatment regimen.

**Conclusion:**

Long-course chemoradiotherapy combined with concurrent tislelizumab in patients with locally advanced low rectal cancer had favorable safety and efficacy, and does not increase the complication rate of surgery. Further study is needed to confirm these results.

## Introduction

Worldwide, colorectal cancer (CRC) is the third most common malignancy (1). Rectal cancer accounts for more than 1/3 of CRC patients. For those with mid-to-low locally advanced rectal cancer (LARC), long-course chemoradiotherapy (CRT) followed by total mesorectal excision (TME) is the standard treatment (2, 3). Generally, the pathological complete response (pCR) rate in conventional CRT was only 10%-20% (4–6). To obtain better oncological outcomes and preservation of organ function, treatment combinations in neoadjuvant therapy have been explored to achieve a higher rate of tumor downstaging.

Immune checkpoint inhibitors (ICIs) have been proven effective in many solid tumors (7–9). In deficient mismatch repair (dMMR) or microsatellite instability-high (MSI-H) colorectal cancer, the ICIs appear favorable clinical benefits (10), while in proficient mismatch repair (pMMR) or microsatellite stable (MSS) subsets, the slight efficacies of ICIs have been reported (11–13). Thus, a combination of CRT and ICIs has been expected to treat such refractory tumors. Preclinical studies have shown a synergistic antitumor effect of this treatment regimen. Radiotherapy promotes the presentation of tumor-derived antigens, upregulates the PD-L1 expression, increases the CD3/CD8 T-cell infiltration, and activates the innate immune pathway (14, 15). These tumor microenvironment remolding effects may enhance the anti-tumor efficacies of ICIs.

A few studies have explored the ICIs combined with CRT in neoadjuvant therapy for LARC. A promising pCR rates of 25%-48.1% were reported with only mild toxicities (16–20). The VOLTAGE-A study added 5 cycles of nivolumab after long-course chemoradiotherapy. A 30% and 60% pCR rates were observed in MSS and MSI-H patients respectively (18). The optimal timing of ICIs use in neoadjuvant therapy is inconclusive. Several studies have shown that ICIs appear to have better synergy with radiotherapy when administered concurrently (21, 22). And the PACIFIC trial demonstrated that the durvalumab given within 14 days after radiation may prolong the overall survival (23). Thus, the PACIFIC-2 aimed to evaluate the benefit of concurrent durvalumab with chemoradiation (NCT03519971). Given these results, we designed this phase II, multicenter, prospective, single-arm trial to evaluate the efficacy and safety of LR-CRT combined with concurrent tislelizumab in patients with LARC (24). In this manuscript, we will report the interim result of this study.

## Materials and methods

This NCRT-PD1-LARC was a prospective, multicenter, single-arm, phase II trial (Clinical trial number: NCT04911517). The study design was described previously (24). To allow patient enrollment in accordance with clinical practice, we undertook a protocol amendment to include patients with mid-to-low locally advanced rectal cancer (0-10cm above anal verge) with cT3-4aN0M0 or cT1-4a N1-2M0 pre-staged by MRI. The major exclusion criteria were congenital or acquired immune deficiency and present or previous active malignancies (except the diagnosis of rectal cancer this time). The protocol and amendments were approved by the ethics committee of Beijing Friendship Hospital, Capital Medical University on March 30^th^, 2021, and February 25^th^, 2022, respectively. The informed consent of study participation was signed before treatment.

### Therapeutic schedule

Eligible patients received long course radiotherapy (50 Gy/25 f, 2 Gy/f, 5 days/week) in the first five weeks and three 21-day cycles of capecitabine (1000 mg/m2, bid, po, day1-14) plus tislelizumab (200 mg, iv.gtt, day8) in the first nine weeks. All patients receive the total mesorectal excision surgery 6-8 weeks after completion of the radiotherapy. Adjuvant therapy regimens after surgery are recommended for chemotherapy according to NCCN guidelines.

Patients are required to complete a baseline assessment prior to treatment, including a complete medical history and physical examination, chest CT, abdominal and pelvic CT, rectal MRI, and colonoscopy. These examinations need to be evaluated again before surgery, and the clinical efficacy is evaluated according to the criteria of the Response Evaluation Criteria In Solid Tumors (RECIST) ver.1.1. Adverse events monitoring is followed up at least every 3 weeks during neoadjuvant therapy. The adverse event was managed according to the consensus recommendations from the Society for Immunotherapy of Cancer (SITC) toxicity management working group.

Postoperative follow-up is performed every 3 months for 1 year and every 6 months thereafter until 5 years after surgery or to death. The complication classification refers to the Clavien-Dindo classification [9].

### Outcomes

The primary outcome was the pathologic complete response (pCR) rate, defined as the proportion of patients with pCR (ypT0N0). The secondary outcomes were as follows (1): The tumor regression was evaluated according to the criteria of the American Joint Committee on Cancer (AJCC) 8th edition. Tumor regression grade (TRG) 0 indicates no residual tumor cells; TRG 1 indicates single or small groups of cells, TRG 2 indicates residual cancer with a desmoplastic response, and TRG 3 indicates minimal evidence of tumor response (2). objective response rate (ORR) is the result of complete response plus partial response rate (3). neoadjuvant rectal (NAR) score was calculated from clinical T stage, pathological T and N stages. A higher score represents a poorer prognosis (4). R0 resection rate was defined as the percentage of the negative margin microscopically (5). Anal preservation rate was defined as the percentage of the patients who received the anal-preserving surgery (6). 3-year local recurrence rate was defined as the percentage of patients who had local recurrence within 3 years after TME surgery (7). 3-year disease-free survival rate is defined as the percentage of patients without recurrence, metastasis, or death within 3 years (8). 3-year overall survival rate was defined as the percentage of patients alive at the 3-year follow-up (9). Safety analysis includes adverse events and postoperative complications. Adverse events were assessed using Common Terminology Criteria for Adverse Events (CTCAE) ver. 4.0, and postoperative complications were assessed using e Clavien–Dindo classification ver. 2.0.

### Statistical analysis

The pCR rate in patients with NCRT was reported to be 15% according to previous studies. We assumed the pCR rate in this trial could increase to 40%. With a one-sided alpha of 5%, power of 80%, and a 10% dropout, 50 patients were needed in this single arm.

Statistical analyses were in progress using the SPSS software (version 22.0). Continuous variables will be presented as means ± standard deviation. Categorical variables will be presented as numbers and percentages. The efficacy and safety analyses were performed in patients treated with at least one dose of tislelizumab and who received radical surgery to obtain the pathological results. Comparisons were performed using Fisher’s exact test or the χ2 test. P values <0.05 were considered statistically significant.

## Results

### Patient characteristics and compliance

At the time of the interim analysis, 38 patients were enrolled in this ongoing study from April 2021 to June 2022. Among them, 26 patients have received neoadjuvant therapy and completed treatment protocol. All patients received the full course of radiotherapy (50Gy) and chemotherapy without dose modification (100%, 26/26). And 24 patients received 3 cycles of tislelizumab (92.3%, 24/26), 2 patients received 2 cycles (first and third cycles) due to adverse events (grade 3 immune checkpoint inhibitor-associated colitis and grade 1 hyperthyroidism). Patient characteristics were shown in **Table 1**.

**Table 1:** Patient characteristics.

Age, years, means (standard)	60.5 (11.8)
Sex, n (%)	
Male	14 (53.8)
Female	12 (46.2)
ECOG performance status, n (%)	
0	16 (61.5)
1	10 (38.5)
Clinical T category, n (%)	
cT2	4 (15.4)
cT3	19 (73.1)
cT4	3 (11.5)
Clinical N category, n (%)	
cN0	12 (46.2)
cN1	9 (34.6)
cN2	5 (19.2)
EMVI, n (%)	
Negative	9 (34.6)
Positive	17 (65.4)
MRF, n (%)	
Negative	22 (84.6)
Positive	4 (15.4)
Distance from primary tumor to anal verge	
Means (standard)	4.9 (2.6)
<5cm, n (%)	12 (46.2)
5-10cm, n (%)	14 (53.8)
Length of tumor lesion, cm, means (standard)	3.7 (1.7)
CEA evaluated, n (%)	9 (34.6)
Time from the end of CRT to radical surgery, weeks, means (weeks)	8.0 (1.7)
Surgery	
Anal-preserving surgery, n (%)	23 (88.5)
Not anal-preserving surgery, n (%)	3 (11.5)

### Surgery

The interval between the completion of radiotherapy and surgery was 8.0 ± 1.7 weeks. A total of 27 patients underwent TME surgery with R0 resection. The anal preservation was 88.5% (23/26). The blood loss was 74.1 ± 41.7 ml. The length of surgery was 222.0 ± 50.6 min. None of the patients had intraoperative complications. Six patients (23.1%) had postoperative complications, including rectovaginal fistula in one patient (grade III), anastomosis leak in one patient (grade II), ileus in two patients (grade II), and deep vein thrombosis in one patient (grade II). The length of the patient’s hospital stay was 12.4 ± 2.9 days. No treatment-related death occurred.

### Efficacy

The interval between the end of radiotherapy and preoperative MRI evaluation was 6.0 ± 1.9 weeks. The efficacy evaluation was shown in **Table 2**. Of the 26 patients, 46.2% (12/26) achieved a complete response, 26.9% (7/26) achieved a partial response, and 26.9% (7/26) achieved stable disease. No patients present with progressive disease. The objective response rate was 73.1% (19/26). All the patients were pMMR subsets, 50% (13/26) patients achieved pCR(ypT0N0), 53.8% (14/26) achieved TRG 0, 26.9% (7/26) patients achieved TRG 1, and 19.2% (5/26) achieved TRG 2. The positive lymph nodes (pN+) were found in 4 patients, of which 2 patients had metastatic lymph nodes and 2 patients had tumor deposits. The NAR scores were 7.2 ± 10.4.

**Table 2:** Efficacy evaluation.

RECIST evaluation, n (%)	
CR	12 (46.2)
PR	7 (26.9)
SD	7 (26.9)
ORR	19 (73.1)
T category, n (%)	
ypT0	14 (53.8)
ypT1	3 (11.5)
ypT2	2 (7.7)
ypT3	7 (26.9)
N category, n (%)	
ypN0	22 (84.6)
ypN1	3 (11.5)
ypN2	1 (3.8)
TRG, n (%)	
0	14 (53.8)
1	7 (26.9)
2	5 (19.2)
pCR, n (%)	13 (50.0)

### Safety

The adverse events that emerged during the neoadjuvant therapy were summarized in **Table 3**. Most treatment-related adverse events were grade 1-2, with only one grade 3 adverse event occurring. The most common treatment-related AEs were fatigue (53.8%), pruritus (42.3%), and radiation enteritis (38.5%). Immune-related adverse events (irAE) occurred in five (19.2%) patients, including one patient with grade 3 immune checkpoint inhibitor-associated colitis, one patient with grade 1 hyperthyroidism, one patient with grade 1 hypothyroidism, one patient with grade 1 hypopigmentation, and one patient with grade 1 bullous pemphigoid. No grade 4 or 5 adverse event occurred in this study.

**Table 3:** Adverse events.

	Patients (n=26)	
Treatment-related AEs, n (%)	Grade I-II	Grade III
Fatigue	14 (53.8)	0
Pruritus	11 (42.3)	0
Radiation Proctitis	10 (38.5)	0
Nausea	8 (30.8)	0
Leukopenia	8 (30.8)	0
Rash	7 (26.9)	0
Diarrhea	7 (26.9)	0
Anemia	6 (23.1)	0
Abdominal pain	5 (19.2)	0
Neutropenia	4 (15.4)	0
Arthralgia	2 (7.7)	0
Alanine transaminase increased	2 (7.7)	0
Chest pain	1 (3.8)	0
Hyperthyroidism	1 (3.8)	0
Hypothyroidism	1 (3.8)	0
Skin depigmentation	1 (3.8)	0
Bullous pemphigoid	1 (3.8)	0
Immune checkpoint inhibitor-associated colitis	0	1 (3.8)
Immune-related AEs, n (%)		
Immune checkpoint inhibitor-associated colitis	0	1 (3.8)
Hyperthyroidism	1 (3.8)	0
Hypothyroidism	1 (3.8)	0
Skin depigmentation	1 (3.8)	0
Bullous pemphigoid	1 (3.8)	0

### Predictive factors analysis for treatment response

The clinical features were examined to analyze the predictive factors for pCR and the results were shown in **Table 4**. The univariate analysis suggested that age <50 years, without pre-treatment carcinoembryonic antigen (CEA) elevation, may be beneficial from the treatment regimen. The pCR rate was 100% (4/4) in young onset rectal cancer patients (age<50) and 40.9% (9/22) in other patients (p=0.03). And the pCR rate was only 11.1% (1/9) in patients with elevated CEA and 70.6% (12/17) in patients without CEA elevation (p=0.004). No significant differences were found in other clinical factors.

**Table 4:** Clinical features of patients with response to the treatment.

	pCR (n=13)	Non-pCR (n=13)	p
Age, years, n (%)			0.030*
<50	4 (30.8)	0 (0)	
≥50	9 (69.2)	13 (100)	
Sex, n (%)			0.431
Male	6 (46.2)	8 (61.5)	
Female	7 (53.9)	5 (38.5)	
CEA level, ng/ml, n (%)			0.004**
<5	12 (92.3)	5 (38.5)	
≥5	1 (7.7)	8 (61.5)	
Differentiation grade			0.095
1	3 (23.1)	0 (0)	
2	9 (69.2)	13 (100)	
3	1 (7.7)	0 (0)	
Clinical T classification, n (%)			0.619
1-2	3 (23.1)	2 (15.4)	
3-4	10 (76.9)	11 (84.6)	
Clinical N classification, n (%)			1
Negative	6 (46.2)	6 (46.2)	
Positive	7 (53.9)	7 (53.9)	
Distance from AV (cm), n (%)			0.431
<5	7 (53.9)	5 (35.5)	
5-10	6 (46.2)	8 (61.5)	
EMVI, n (%)			0.680
Negative	5 (38.5)	4 (30.8)	
Positive	8 (61.5)	9 (69.2)	
MRF, n (%)			0.277
Negative	10 (76.92)	12 (92.3)	
Positive	3 (23.08)	1 (7.7)	
Radiotherapy-surgery interval, weeks, n (%)			0.216
≥7	7 (53.9)	10 (76.9)	
<7	6 (46.2)	3 (23.1)	

*p<0.05, **p<0.01.

## Discussion

While the ICIs have shown promise in dMMR/MSI-H rectal cancers, they are generally ineffective in pMMR/MSS rectal cancers (11). However, CRT combined with ICIs is considered to have a good synergistic effect. A more immunologically active microenvironment was found after CRT: an increase in CD8+ T-cell infiltration and upregulated PD-L1 expression (14, 15). In this rationale, an addition of ICIs may enhance the anti-tumor effect. The clinical efficacy of chemoradiotherapy combined with immunotherapy has been proven effective in many tumors (25–31), particularly in non-small cell lung cancer, this regimen has rarely been reported as neoadjuvant therapy in rectal cancer. To our knowledge, our study is the first to propose a neoadjuvant therapy of a concurrent long-course CRT and ICIs combination and achieved a high pCR rate of 50% in pMMR LARC patients with no serious adverse events occurring. The pCR rate reached 50%, much higher than the 10%-20% of traditional neoadjuvant therapies (4–6) and also higher than the 25%-46.2% of other studies using ICI combined with CRT (16–20).

This study reported a fairly good tumor regression efficacy. The CR and ORR reached 46.2% and 73.1%, respectively. The improvement of CR rate will be of great significance to the organ preservation of LARC patients after radiotherapy and chemotherapy through “Watch and Waite” policy or selective local excision. In the Maas study, 192 patients treated with traditional chemoradiotherapy, 21 patients (10.9%) achieved clinical complete regression and underwent organ preservation through “Watch and Waite” policy (32). In the ACCORD12/PRODIGE 2 study, 201 LARC patients were evaluated for clinical tumor response after neoadjuvant therapy, and ths score was: complete response: 8%; partial response: 68%; stable: 21%; progression: 3%. The CR rate of CAPOX+radiotherapy group was higher than that of capecitabine+radiotherapy group (9.3% vs 6.7%) (33). Our study reported a similar ORR rate, but a significantly higher CR rate (46.2%). Therefore, it is promising to further study and explore organ preservation after chemoradiotherapy combined with immunotherapy.

Various combination regimens of CRT and ICIs have been reported. In the VOLTAGE-A study, 5 cycles of nivolumab followed by CRT resulted in a 30% pCR rate in pMMR rectal cancer patients (18). It is suggested that the use of ICIs in advance in the course of radiotherapy and chemotherapy may achieve a better synergistic effect. The dose scheduling with concurrent but not sequential therapy was also proved to be effective in tumor regression in preclinical studies (22). The neoadjuvant therapy of adding ICIs to the regimen of short-course radiotherapy combined with CAPOX or FOLFOX also achieved favorable results, WUGO-001 and AVERECTAL studies reported the pCR rate of 48.1% and 37.5% respectively (17, 34). However, the NRG-GI002 study reported a similar pCR rate comparing the concurrent long-course CRT plus pembrolizumab and long-course CRT alone after FOLFOX induction (31.9% versus 29.4%) (16). This suggests that chemotherapy may be more effective as a consolidation regimen rather than an induction regimen.

It is critical to screen the beneficiaries of this neoadjuvant strategy. The VOLTAGE-A study showed that the elevated expression of PD-L1 and CD8/eTreg ratio before treatment were more likely to benefit from the immunotherapy. Among patients with PD-L1 (TPS) ≥ 1%, 75% of patients achieved pCR, while in the PD-L1 (TPS) <1% group, only 17% of patients achieved pCR (18). By analyzing the clinical features, we found CEA was a negative predictor of tumor response. The pCR rate of 11.1% was achieved in patients with CEA elevating compared with 70.6% in those without CEA elevating. This was consistent with previous studies that pre-treatment CEA was inversely correlated with pCR (35, 36). Another predictive factor that we identified was age less than 50 years. These young-onset rectal patients have a promising response to the neoadjuvant treatment with a 100% (4/4) pCR rate. Certain pathological characteristics were reported in colorectal patients less than 50 years, including poor tumor differentiation and low tumor-infiltrating lymphocytes, which were considered to have poor anti-tumor immune response (37). However, this condition may be reversed under the regimen of chemoradiotherapy combined with immunotherapy.

This manuscript reported the interim result of this study. The limitations include the small sample size, single-arm design, and no long-term survival data. Despite this, the result of the high pCR rate was encouraging. We will continue to complete study enrollment and follow-up. Biomarkers will also be analyzed using pre and post-treatment tumor samples. Further large randomized controlled phase III study is worth to

In conclusion, long-course chemoradiotherapy combined with tislelizumab followed by TME surgery showed a favorable pCR rate and well-tolerated toxicities in pMMR rectal cancer patients. Patients with no CEA elevation or young-onset rectal cancer are more likely to benefit from this treatment regimen. Further large-scale randomized controlled studies are required to confirm this result.

## Data availability statement

The raw data supporting the conclusions of this article will be made available by the authors, without undue reservation.

## Ethics statement

Written informed consent was obtained from the individual(s) for the publication of any potentially identifiable images or data included in this article.

## Author contributions

HY and ZZ designed this study; JG, ZY, XZ, ZB, WD, QW, JH, AL, GL, YS, DK enrolled and managed patients, and collected the data. JZ reviewed the MRI. GC and RX were responsible for pathological assessment. YL provided the administrative support. JG, XZ, ZY drafted the manuscript and all authors reviewed. HY and ZZ had full access to all the data in the study and had final responsibility for the decision to submit for publication. The final version was approved to be submitted by all authors. HY and ZZ are guarantors of the work.
